# Clinical Pharmacy Education in Japan: Using Simulated Patients in Laboratory-Based Communication-Skills Training before Clinical Practice

**DOI:** 10.3390/pharmacy6020049

**Published:** 2018-06-01

**Authors:** Rie Kubota, Kiyoshi Shibuya, Yoichi Tanaka, Manahito Aoki, Megumi Shiomi, Wataru Ando, Katsuya Otori, Takako Komiyama

**Affiliations:** Research and Education Center for Clinical Pharmacy, School of Pharmacy, Kitasato University 5-9-1 Shirokane, Minato-ku, Tokyo 108-8641, Japan

**Keywords:** pharmacy education, simulated patient, preliminary education, communication skill

## Abstract

The Japanese pharmaceutical curriculum was extended from four to six years in 2006. Students now receive practical communication-skills training in their fourth year, before progressing to train in hospital and community pharmacies in their fifth year. Kitasato University School of Pharmacy, Tokyo, had established a program to meet these aims before the 2006 guidance. In the present study, we discuss and evaluate the features of this communication-skills training program. This study enrolled 242 fourth-year pharmacy students at Kitasato University. Students filled out a questionnaire survey after completing the laboratory element of their undergraduate education. As part of training, students were asked to obtain patient data from a model medical chart, before performing simulated patient interviews covering hospital admission and patient counseling. These simulations were repeated in a small group, and feedback was provided to students by both the simulated patient and the faculty after each presentation. It was found that students were able to develop their communication skills through this approach. Thus, an effective system of gradual and continuous training has been developed, which allows students to acquire clinical and practical communication skills.

## 1. Introduction

In 2006, the Japanese pharmaceutical curriculum was extended from four to six years [1], with each pharmacy college required to implement a new core curriculum [2,3]. Since then, students have received preliminary education and practical training in laboratory settings during their fourth year of study. Following this, students are assessed for knowledge, skill and attitude through common achievement tests, computer-based testing, and objective-structured clinical ability examinations (OSCEs) [4]. If successful, a student progresses to the fifth year when they engage in training at hospital and community pharmacies for eleven weeks each.

Kitasato University School of Pharmacy, a private school in Japan, has established fundamental and clinical education early. Currently, a six-year course is available through the Pharmaceutical Department and a four-year course is available through the Life Sciences and Drug Development Department. However, both the courses are undergoing constant evolution to address the changing needs of clinical pharmacy. An example of this is the drive to provide a more advanced pharmacist education to solve medical problems using basic research. To complement this, we have introduced several clinical programs to provide early exposure and preliminary practical education in the six-year pharmaceutical course. The required practical hospital training is provided at four hospitals affiliated to Kitasato University, but fourth-year students receive preliminary education in a laboratory setting over a four-month period. During this preliminary period, they learn about drug information evaluation, physical assessment, dispensing, intravenous preparation and patient counseling. 

In laboratory practice, that is, one component of didactic curriculum, hands-on skill-based labs, simulated inpatient interviews and counseling were used to conduct training and develop an understanding of the patients’ background and feelings. Many authors have demonstrated that the simulation of clinical teaching was effective [5,6,7]. Simulation is described as substitution of a real patient for trained–simulated patients enacting a patient experience [5,6,7]. Medicine and nursing schools use patient simulation to teach a broad range of clinically related skills [8,9]. Additionally, patient simulation (role play) is one of the teaching methods in pharmacy education. Moreover, pharmacy practice-related skills including communication have been recently expanded and incorporated in such training. Rickles reported that the use of simulated patients in teaching communication skills to pharmacy students was effective [7].

The aims of this study were as follows: (1) to introduce the advanced features of an element of this education program that seeks to develop communication and problem-solving skills; (2) to evaluate the perceived effectiveness of this education among students.

## 2. Materials and Methods 

### 2.1. Objectives and Study Design

This study was conducted among 242 fourth-year pharmacy students at Kitasato University. The survey focused on the effects of the simulated patient, and requested that students evaluated the teaching method and gave reasons for their responses. The students filled out the questionnaires after their classes. The questions included were as follows: (1) was it effective that simulated patient played a role in simulation? (Multiple responses were chosen from five listed reasons.); (2) was the teaching method wherein the faculty added step-by-step comments within a group effective? (Multiple responses were chosen from four listed reasons.)

### 2.2. Overview of the Preliminary Education: Patient Interview and Counseling

**Figure 1:**
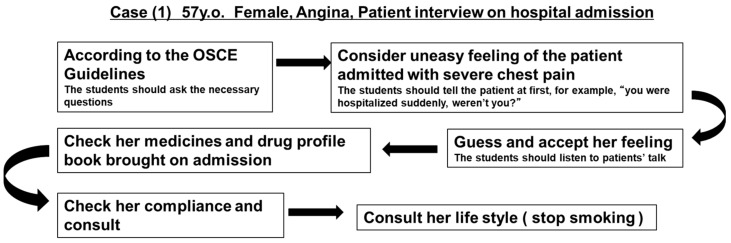
A representative feedback process followed by the faculty for patient simulations.

This laboratory work was delivered to students over three days. For each session, 30 students were categorized into four groups, and one faculty member and one simulated patient were included per group, covering a different case each (see Table 1). On day one, the students were asked to obtain patient data from a model medical chart, confirm the patient’s background, and list the patient’s problems in an appropriate format. On day two, they simulated a patient interview covering hospital admission. For this, we used a classroom and set up four beds in it. Simulated patients sat on the beds and one student interviewed them. The other students, who were observers, surrounded the bed. During these, each student engaged in a simulated interview in their small group, before the other students who had observed them gave their feedback. Also, the simulated patient provided feedback about behaviors after each presentation, and the faculty staff provided feedback about additional points (see Figure 1 for an example). In this way, communication in the simulated interviews was improved in a step-by-step manner within each group. One representative from each group then simulated the patient interview in front of the whole class. On day three, the students simulated patient counseling regarding medicines for internal use, including intravenous therapy, using the same method that was followed on day two. Table 1 summarizes the details of the simulated patients and Figure 1 outlines the feedback process and concrete comments from the faculty. The simulated patients belong to the Institute of My Informed Consent (association for dispatching simulated patients), and have been trained to portray a character or a patient problem as described in a scripted case scenario.

## 3. Results

**Figure 2:**
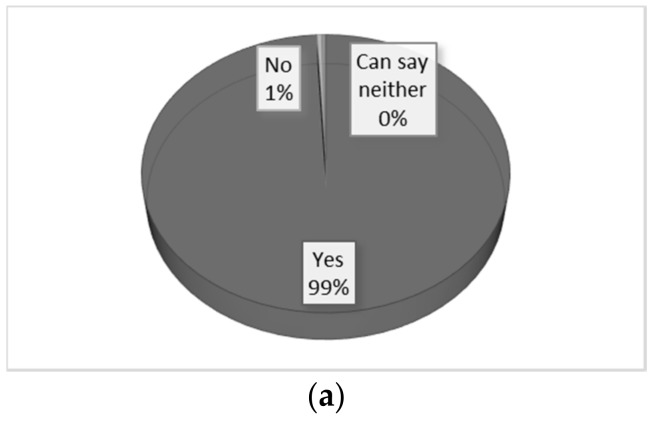

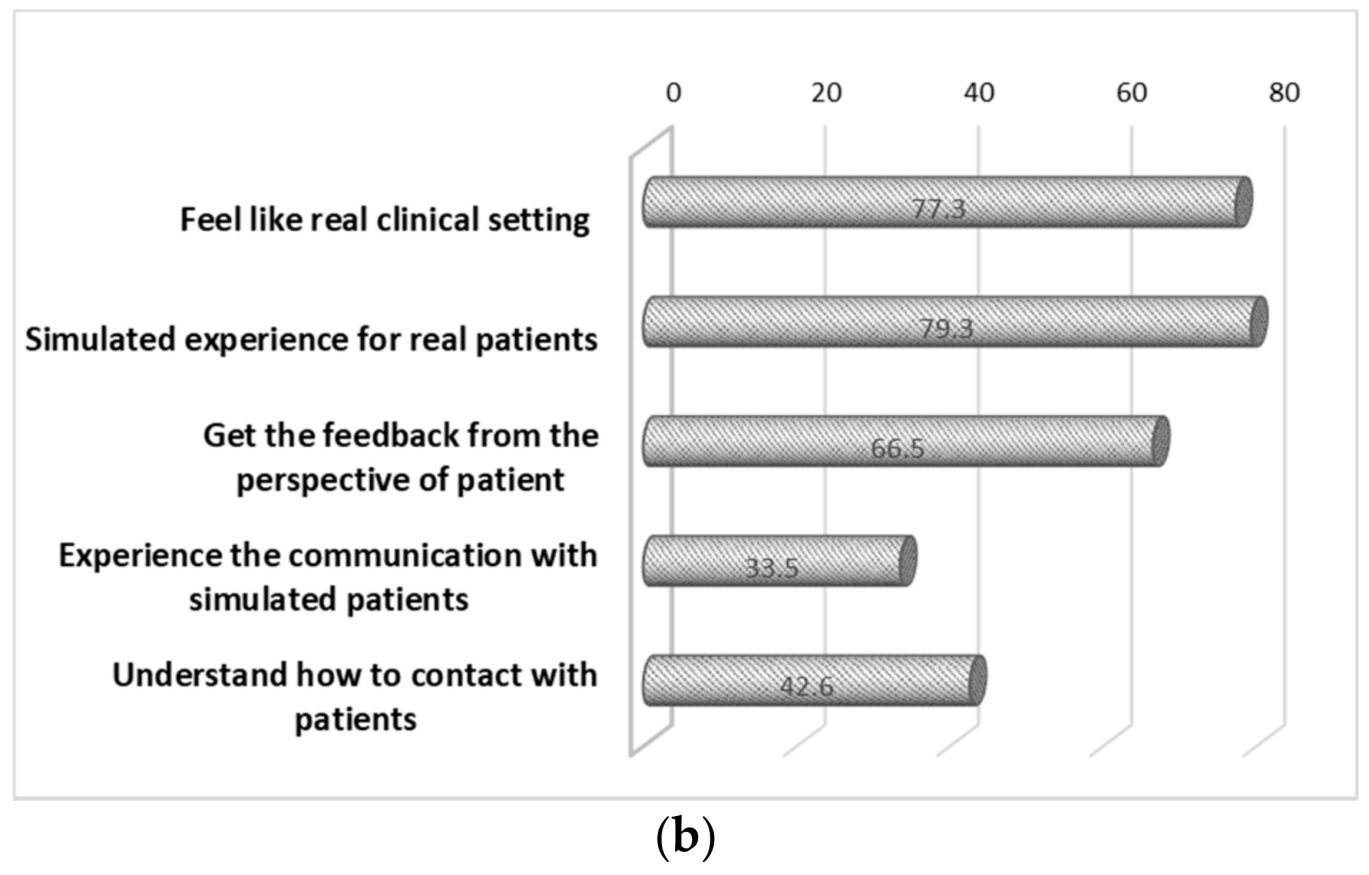
The effectiveness of using a simulated patient for interview and counseling. (**a**) Response to the question “was it effective that simulated patient played a role in simulation?” (**b**) The reasons for the response given to the question in (**a**); multiple responses were permitted.

**Figure 3:**
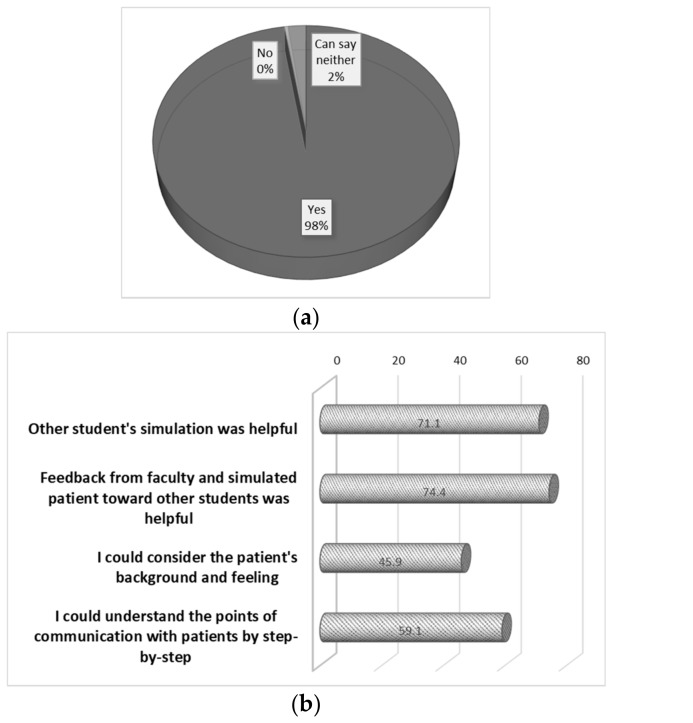
Evaluation of the simulation method for teaching about patient interviews and counseling. (**a**) Response to the question “was the teaching method wherein the faculty added step-by-step comments within a group effective?” (**b**) The reasons for the response given to the question in (**a**); multiple responses were permitted.

Questionnaires were completed by all 242 students (response rate, 100%). Regarding whether it was considered effective to use a simulated patient, 99% responded positively. Frequent explanations for the high effectiveness (Figure 2) were that “they could simulate the experience just like with a real patient” (79.3%), “they could raise the presence” (77.3%), and “they could get the feedback from the perspective of patient” (66.5%). In response to whether teaching by faculty staff was effective when adding comments in the step-by-step group discussion, 98% of students answered positively. Among the most frequent reasons for this (Figure 3) were that “feedback from the faculty and simulated patient to peers was useful” (74.4%), “simulations performed by peers were helpful” (71.1%), and “communication points could be understood through the step-by-step analysis” (59.4%).

## 4. Discussion

The six-year education that was introduced in 2006 aimed to train pharmacists to have not only fundamental scientific knowledge but also a humanistic approach, medical ethics, refinement as a medical specialist, and problem-solving and practical skills.

Practical clinical training is required for at least six months in both hospital and community pharmacies before taking the national examination. This is in line with the 2010 recommendation by the Ministry of Health, Labor and Welfare in Japan, which stated that interprofessional collaboration with other healthcare professionals should be promoted [10]. Indeed, it is considered that the pharmacist should be an expert and authority in medications, being best-placed in medical teams to ensure patient safety and improve medical therapy. The six-year core curriculum therefore aims to improve medical knowledge, skills and attitudes, including key attributes like problem-solving and communication skills [3]. At Kitasato University School of Pharmacy, such programs had already been introduced before 2006, using simulated patients to improve communication skills. This was used as a preliminary education program before students entered clinical training at hospital and community pharmacies. Based on our experience and the results presented, the described training for inpatient interview and counseling is effective.

Hospital pharmacists should be skilled in interviewing patients on hospital admission, for medication counseling, and before discharge. When patients are admitted to hospital, pharmacists should ask about symptoms, disease progression, medication history, and experience of adverse events or allergy. However, as with any other healthcare professional, pharmacists should also consider the patient’s background and feelings. To meet this requirement, each student must now have their skills and attitudes evaluated in OSCEs before they can continue training at hospital and community pharmacies [4]. Fifth-year students can then practice medication monitoring, interviews and counseling with the support of a trainer during their clinical experience at a hospital pharmacy [11].

In laboratory practice, simulated inpatient interviews and counseling were used to provide training and develop an understanding of the patients’ backgrounds and feelings, paying attention to the requirements of OSCEs [4]. Simulation is an adaptable teaching method and can be used to incorporate a range of skills and knowledge in students pursuing pharmacy [12]. Barrow defined the term simulated patient in 1964, and reported that using a simulated patient instead of actual patients was useful as a teaching and assessment tool in the classroom [13]. Today, several pharmacy schools in Japan use simulated patients to deliver communication-skills education [14]. In the present cases, the simulated patients had received specialized training for each scenario. Most of the students (99%) reported that simulated patients were effective. The students felt like they communicated with real patients, and the simulation created an authentic environment. Moreover, simulated patients can repeat the clinical scenarios and cases can be offered to them on demand [15]. Perhaps most importantly, the students indicated that using these simulated patients was an effective method of role play and that they felt like they were communicating with real patients. Additionally, simulated patients can enhance patient safety because students practice clinical skills without involving actual patients, thereby reducing risk to actual patients [16]. Feedback from the simulated patients was also highly valued and was considered to help the students acknowledge the patient’s perspective.

The communication-skills training in this study was delivered in large groups of 40 students per class because of staffing limitations. To deliver an effective teaching method, the simulations were then repeated in small groups of seven or eight students, in which students received feedback from their peers, the simulated patient and the faculty. Most students (98%) reported that this method was effective. Although students did not repeat simulations, they did feel that it was useful to observe their peers and to share in the feedback process. They also felt that the feedback from patients helped them to better understand communication dynamics. Simulated patients were closely matched to the patient scenario to enhance the clinical situation simulation. Additional feedback from the faculty staff supported learning from peers and patients, allowing them to improve in a step-by-step manner within a group. This step-by-step approach is important for students to improve their knowledge and communication skills.

All of the students could pass the part of OSCEs related to simulated inpatient interviews and counseling, and continue the clinical training at the hospitals after this experience. The teaching method advocated in this study, based on small-group discussion of simulated patient interviews, has several key requirements. Before training in the communication-skills laboratory, the details of interviews must be arranged and agreed with the simulated patient to ensure consistency and that learning objectives are met. During this, there is a need to ensure that the scenario includes relevant aspects of how the patient feels and his or her background, ensuring that questions are asked to obtain the patient’s viewpoint during feedback. Faculty members must also work in collaboration with the simulated patient and share in the standardization of the teaching method and its goals.

## 5. Conclusions

The change in curriculum in 2006 requires that students engage in a gradual and continuous program to acquire clinical and practical skills. Overall, the available evidence suggests that simulated patients are suitable for use in communication-skills training. This study was limited to evaluate the efficacy of this training. We would further like to evaluate whether this clinical pharmacy training contributes to clinical practice in a hospital setting.

## Figures and Tables

**Table 1:** Details of simulated patients.

Case	Patient’s Background	Points of Interview	Points of Counseling
57-year-old female with angina	Patient admitted with severe chest pain Non-adherence	Patient’s adherence Consider patient’s feelings	Using nitroglycerin and isosorbide dinitrate tape Coping with non-adherence Advice to quit smoking
60-year-old female with diabetes	Patient had sudden episodes of increased blood sugar Patient took health food Patient was reluctant to undergo insulin therapy	Check the lifestyle and health food intake Consider patient’s feelings	Explaining insulin and oral hypoglycemic drug Counseling to adopt healthy lifestyle
65-year-old male with ureteral stone	Severe pain Anxiety for drug intake due to adverse effect	Consider patient’s feelings	Explain about the change of drug
68-year-old male with gallstone	Surgery for gallstone Discontinued anticoagulant agent before surgery Anxiety for surgery	Check discontinued drug Consider patient’s feeling before surgery	Consider patient’s condition after surgery
